# Effects of supplementing with an 18% carbohydrate-hydrogel drink versus a placebo during whole-body exercise in −5 °C with elite cross-country ski athletes: a crossover study

**DOI:** 10.1186/s12970-019-0317-4

**Published:** 2019-10-26

**Authors:** Stefan Pettersson, Fredrik Edin, Linda Bakkman, Kerry McGawley

**Affiliations:** 1 Center for Health and Performance, Department of Food and Nutrition, and Sport Science, University of Gothenburg, Gothenburg, Sweden; 2 Swedish Olympic Committee, Stockholm, Sweden; 3 Swedish Winter Sports Research Centre, Department of Health Sciences, Mid Sweden University, Östersund, Sweden

**Keywords:** Biathlete, Cold, Double-poling, Endurance, Roller-skiing, Sex differences, Stabile isotopes, Substrate utilization, World-class

## Abstract

**Background:**

Whilst the ergogenic effects of carbohydrate intake during prolonged exercise are well-documented, few investigations have studied the effects of carbohydrate ingestion during cross-country skiing, a mode of exercise that presents unique metabolic demands on athletes due to the combined use of large upper- and lower-body muscle masses. Moreover, no previous studies have investigated exogenous carbohydrate oxidation rates during cross-country skiing. The current study investigated the effects of a ^13^C-enriched 18% multiple-transportable carbohydrate solution (1:0.8 maltodextrin:fructose) with additional gelling polysaccharides (CHO-HG) on substrate utilization and gastrointestinal symptoms during prolonged cross-country skiing exercise in the cold, and subsequent double-poling time-trial performance in ~ 20 °C.

**Methods:**

Twelve elite cross-country ski athletes (6 females, 6 males) performed 120-min of submaximal roller-skiing (69.3 ± 2.9% of $\dot{\mathrm{V}}$O_2_peak) in −5 °C while receiving either 2.2 g CHO-HG·min^− 1^ or a non-caloric placebo administered in a double-blind, randomized manner. Whole-body substrate utilization and exogenous carbohydrate oxidation was calculated for the last 60 min of the submaximal exercise. The maximal time-trial (2000 m for females, 2400 m for males) immediately followed the 120-min submaximal bout. Repeated-measures ANOVAs with univariate follow-ups were conducted, as well as independent and paired t-tests, and significance was set at *P* < 0.05. Data are presented as mean ± SD.

**Results:**

Exogenous carbohydrate oxidation contributed 27.6 ± 6.6% to the total energy yield with CHO-HG and the peak exogenous carbohydrate oxidation rate reached 1.33 ± 0.27 g·min^− 1^. Compared to placebo, fat oxidation decreased by 9.5 ± 4.8% with CHO-HG, total carbohydrate oxidation increased by 9.5 ± 4.8% and endogenous carbohydrate utilization decreased by 18.1 ± 6.4% (all *P* < 0.05). No severe gastrointestinal symptoms were reported in either trial and euhydration was maintained in both trials. Time-trial performance (8.4 ± 0.4 min) was not improved following CHO-HG compared to placebo (− 0.8 ± 3.5 s; 95% confidence interval − 3.0 to 1.5 s; *P* = 0.46). No sex differences were identified in substrate utilization or relative performance.

**Conclusions:**

Ingestion of an 18% multiple-transportable carbohydrate solution with gelling polysaccharides was found to be well-tolerated during 120 min of submaximal whole-body exercise, but did not improve subsequent maximal double-poling performance.

## Background

It is well recognized that supplementing with carbohydrate (CHO) solutions during prolonged (i.e., > 2 h) moderate- to high-intensity exercise improves endurance capacity and performance [1]. The performance-enhancing mechanisms attributed to CHO ingestion during exercise include sparing of liver glycogen, maintenance of euglycemia and high rates of CHO oxidation, which enable maintenance of high exercise intensities. Since intestinal absorption is likely the main rate-limiting step in exogenous CHO delivery to muscle during exercise, previous studies have attempted to optimize absorption rates by saturating different intestinal transport mechanisms. For example, it is generally agreed that the maximal rate of CHO absorption is ~ 1 g·min^− 1^ when only glucose or glucose polymer solutions are fed in excess of 1.2 g·min^− 1^ during exercise [2]. However, by ingesting mixtures of glucose and fructose, which are absorbed by different transporters (SGLT1 and GLUT-5, respectively), exogenous CHO oxidation rates have been demonstrated to increase 1.2- to 1.7-fold during prolonged exercise. Research also suggests reductions in gastrointestinal (GI) discomfort following intake of multiple-transportable CHO mixtures compared to isocaloric glucose-only intakes [2–4].

A recent innovation for providing CHO during exercise is the inclusion of alginate and pectin, two polysaccharides with gelling properties, to a sports beverage [5]. In a field study of 16 elite long-distance runners, Sutehall et al. [6] reported high tolerability of a highly concentrated (30%) maltodextrin-fructose solution with additional alginate during a training run (25.1 km, average pace ~ 3.5 min·km^− 1^). The authors speculated that this high tolerability was related to the encapsulation of the liquid CHO under the acidic conditions of the stomach, which forms a hydrogel. Perhaps less beneficial in terms of athletic performance is that alginate, added in sufficient amounts for strong gelling in the stomach, can attenuate the glucose response, predominately by slowing the rate of gastric emptying following ingestion of a meal or a CHO-containing drink [7, 8].

The majority of studies investigating the effects of CHO intake, exogenous CHO oxidation and endurance performance have used cycling or running protocols. This presents practical limitations when extrapolating the results to other modes of exercise. For example, energy intake requirements are likely higher during competitive cross-country (XC) skiing compared to other endurance sports, due to the simultaneous activation of large upper- and lower-body muscle masses and the resulting high-energy turnover and demand for CHO availability. This has been illustrated by studies showing leg muscle glycogen to be depleted by ~ 50–100% following 10- and 50-km XC ski races, and reductions in stored arm glycogen to ~ 30% of pre-exercise levels following 1 hour of intense diagonal-style XC skiing [9, 10]. Furthermore, XC skiing is often performed in low ambient temperatures, which has been shown to increase CHO oxidation when compared to exercising in warmer environments [11].

To date, only two studies have investigated the effects of CHO intake on XC skiing performance. Viinamäki and colleagues [12] found a non-significant trend for improved 50-km race performance following ingestion of 2.75 g·CHO min^− 1^ compared to a volume-matched 2.5% glucose solution providing 0.3 g CHO·min^− 1^. More recently, Stocks et al. [13] reported no significant effects of ingesting a multiple-transportable CHO solution at different ingestion rates (1.2 versus 2.4 g CHO·min^− 1^) or frequencies (every 5 versus 15 km) during a 30-km simulated XC skiing race. These studies are, however, limited by the absence of a controlled placebo trial and the measurement of exogenous CHO oxidation, which makes it difficult to draw conclusions about the impact of CHO supplementation on performance, as well as the determinants of fatigue related to substrate utilization during XC skiing.

Most studies measuring the ergogenic effects of CHO supplementation have been performed with male participants [14]. Among the few studies investigating females most [15–17], but not all [18] demonstrate performance benefits with CHO compared to a placebo. Mediated primarily by the ovarian hormone estrogen, females have been shown to oxidize more fat and less CHO relative to lean body mass (LBM) at a given submaximal exercise intensity in a fasted state, compared with males [19]. However, these sex differences in substrate utilization seem to diminish following CHO supplementation. For example, studies using isotopic carbon-13 (^13^C) labelling techniques have revealed no significant sex differences in the relative contribution from exogenous oxidation of CHO when ingesting a single-transportable CHO (glucose) [20–23]. However, exogenous CHO oxidation using a multiple transportable CHO solution has not been directly compared between females and males.

The first aim of the present study was to compare, in a group of elite athletes, the rates of substrate oxidation, blood lactate and glucose responses, as well as subjective GI discomfort and rating of perceived exertion (RPE), during 120 min of submaximal diagonal-style roller-skiing in −5 °C with the ingestion of either an 18% CHO hydrogel drink (CHO-HG) or a non-caloric placebo (PLA). The second aim was to compare the effects of a subsequent double-poling time-trial in ~ 20 °C (TT) on performance, blood lactate, blood glucose, GI and RPE responses. Due to the composition of the sample group, an additional aim was to compare the differences in male and female responses to submaximal and maximal XC ski exercise with ingestion of CHO-HG and PLA. It was hypothesized that ingestion of the CHO-HG drink would be well tolerated, would lead to sparing of endogenous CHO and would be associated with improved double-poling performance in both males and females.

## Methods

### Participants

Twelve elite XC ski athletes (6 females, 6 males) participated in the study (Table 1). Nine of the participants (4 females, 5 males) were members of the Swedish national biathlon team and five of those (3 females, 2 males) were medallists at the PyeongChang 2018 Winter Olympic Games. The remaining three participants (2 females, 1 male) were elite XC skiers, one of whom was a multiple Olympian and long-distance World Champion. Two of the six female participants used a monophasic oral contraceptive pill and one used a hormonal spiral. The other three females did not use hormonal contraception and completed their first experimental trial on day 4, 20 or 23 of their menstrual cycle. All participants completed their second experimental trial within 7 days. Data was collected during the summer pre-season phase and training data (amount, type and intensity) during the 4 weeks preceding the experimental trials are presented in Table 1. All athletes gave their written informed consent prior to participating in the study. Test procedures were performed following the Declaration of Helsinki and approved by the local ethics committee of Gothenburg University (Dnr: 672–17).

**Table 1:** Descriptive and training characteristics of the 12 participants (mean ± SD)

		Females (*n* = 6)	Males (*n* = 6)
Age	years	24.8 ± 5.3	25.6 ± 4.7
Height	cm	167.7 ± 8.0	181.1 ± 3.1^*^
Body mass	kg	62.1 ± 6.4	75.7 ± 5.5^*^
Lean body mass	kg	46.3 ± 5.2	62.3 ± 2.8**
Body fat	%	21.4 ± 0.9	13.5 ± 4.7^*^
$\dot{\mathrm{V}}$O_2_peak	L·min^−1^	3.6 ± 0.5	5.3 ± 0.4**
	mL·kg^− 1^·min^− 1^	59.9 ± 2.6	69.1 ± 2.9**
	mL·kg LBM^− 1^·min^− 1^	81.1 ± 3.7	85.3 ± 6.7
Weekly training load (h)^a^		17.5 ± 3.4	19.7 ± 2.2
*Of which:*			
Endurance exercise (h)	≤ 75% HR	12.8 ± 3.9	12.9 ± 4.1
	75–80% HR	1.6 ± 1.0	3.0 ± 2.9
	80–85% HR	0.9 ± 0.4	1.4 ± 0.7
	≥ 85% HR	0.9 ± 0.7	0.8 ± 0.5
Gym-based strength training (h)		1.2 ± 0.8	1.9 ± 0.9

*LBM* lean body mass, *HR* heart rate

^a^During the four weeks preceding the experimental trials

*Significantly different from females, *P* < 0.05

**Significantly different from females, *P* < 0.001

### Study overview

Participants attended the laboratory on five separate occasions, firstly completing body composition measurements then a preliminary exercise trial, a familiarization and two experimental trials. They were instructed to abstain from alcohol and to perform only moderate-intensity exercise the day before the preliminary exercise and experimental trials. The preliminary exercise trial was performed in order to determine the submaximal work- $\dot{\mathrm{V}}$O_2_ relationship, $\dot{\mathrm{V}}$O_2_peak and maximal heart rate (HR_max_). The familiarization trial was used to identify the individual treadmill speeds required to elicit ~ 70% of $\dot{\mathrm{V}}$O_2_peak, as well as to familiarize the athletes with the temperature, equipment and procedures used during the two experimental trials. The two experimental trials were conducted using a double-blind, randomized, crossover design and consisted of 120 min of submaximal diagonal-style roller-skiing in −5 °C, followed immediately by a maximal double-poling performance test in ~ 20 °C using a ski ergometer. Immediately prior to and throughout the 120-min submaximal exercise bout participants received either a ^13^C-enriched 18% carbohydrate-hydrogel drink (CHO-HG) or a placebo (PLA), which was designed to mimic the texture and sweetness of the CHO-HG drink.

### Body composition

Following an overnight fast, participants were weighed (Seca 764, Hamburg, Germany) in their underwear and body composition was assessed by dual-energy X-ray absorptiometry (iDXA; GE Medical Systems, Madison, WI, USA). The iDXA was calibrated according to the manufacturer’s guidelines before each measurement. Total lean and relative fat percentages were analyzed using enCore software (version 16.10).

### Preliminary exercise trial

Participants performed an incremental test consisting of four to five, 4-min submaximal stages on a motor-driven treadmill (Rodby Innovation AB, Vänge, Sweden) using the skate roller-skiing technique. The roller skis (Pro-Ski S2, Sterners, Dala-Järna, Sweden) were pre-warmed in order to standardize the rolling resistance and participants wore a safety harness around the waist connected to an automatic emergency brake above the treadmill. The submaximal test was followed by 4 min of active recovery, 5 min of passive recovery and a 5-min active re-warm-up including three, 10–15-s self-paced high-intensity intervals. The maximal test followed, which consisted of 900-m and 1000-m self-paced time-trials for the females and males, respectively. Pulmonary gas exchange was measured throughout both the submaximal and maximal tests using a metabolic cart (AMIS 2001 model C, Innovision A/S, Odense, Denmark) equipped with a flowmeter. The gas analyzers were calibrated with a high-precision two-component gas mixture of 16.0% O_2_, and 4.0% CO_2_ (Air Liquide, Kungsängen, Sweden). Calibration of the flowmeter was performed with a 3 L air syringe (Hans Rudolph, Kansas City, MO, USA) for low, medium and high flow rates. $\dot{\mathrm{V}}$O_2_, $\dot{\mathrm{V}}$CO_2_, and ventilation rate were monitored continuously, and $\dot{\mathrm{V}}$O_2_ values were calculated from 10-s epochs and reported as 30-s averages.

### Familiarization trial

A familiarization trial was used to acquaint the participants with the test procedures and to determine individual treadmill speeds for the subsequent experimental trials. Participants performed a continuous 32-min submaximal effort in an environmental chamber set to −5 °C. To control ambient conditions the chamber utilized a hypoxia controller (Hypoxico, New York, USA), which was set to ‘sea level’ (20.9% O_2_), and a customized air-conditioning system controlling room temperature with a stated precision of ±0.5 °C. The exercise was performed using the diagonal-stride technique and classic roller-skis (Pro-ski C2, Sterners, Dala-Järna, Sweden) on a motor-driven treadmill (Rodby Innovation AB, Vänge, Sweden) fixed at a 5° incline. The starting speed was based on the submaximal work- $\dot{\mathrm{V}}$ O_2_ relationship derived from the preliminary exercise trial, with continuous adjustments made to the treadmill speed until heart rate (HR) stabilized at an intensity corresponding to ~ 70% of $\dot{\mathrm{V}}$O_2_peak (equivalent to mean ± standard deviation [SD] 82 ± 3% of HR_max_). Treadmill speed was reduced to 4 km·h^− 1^ for 30 s after 10 min and every 20 min thereafter, as well as for 90 s after 20 min and every 20 min thereafter, during the familiarization and experimental trials. The 30-s recovery periods allowed participants to change sub-technique and therefore movement pattern, which is not usually fixed for long durations during XC skiing and was considered a potential injury risk. The 90-s recovery periods again allowed for this precautionary alteration in movement pattern, but also enabled fingertip blood sampling, psychometric data collection and consumption of the drink solution.

Following the 32-min submaximal exercise bout participants exited the environmental chamber, were given the opportunity to empty their bladder, then removed any surplus clothing and changed from ski boots to indoor training shoes. They then completed the TT in ~ 20 °C using a ski ergometer (SkiErg, Concept2, Morrisville, VT, USA). The reasons for moving to room temperature were twofold: 1. The display on the ski ergometer did not function reliably at sub-zero temperatures; 2. The coaches and athletes were uncomfortable performing maximal exercise at sub-zero temperatures at this point in the season, due to the increased risk for harm to the airways associated with high ventilation rates in the cold. The TT was a self-paced, double-poling performance test lasting 2000 m for females and 2400 m for males, with the flywheel resistance set at 6 and 8, respectively. The protocol was designed to simulate the muscular work and physiological responses involved in a biathlon competition, which consists of three or five high-intensity bouts of skiing, each lasting up to 8 min [24]. Participants were instructed to complete the set distance as quickly as possible and no encouragement or feedback was provided, except that distance remaining was visible throughout. Given the elite level of the athletes, and the regularity with which they perform intensive double-poling ergometer exercise as a part of their habitual training, one familiarization trial was considered sufficient to establish reproducibility during the subsequent experimental trials.

### Experimental trials

**Fig. 1:**
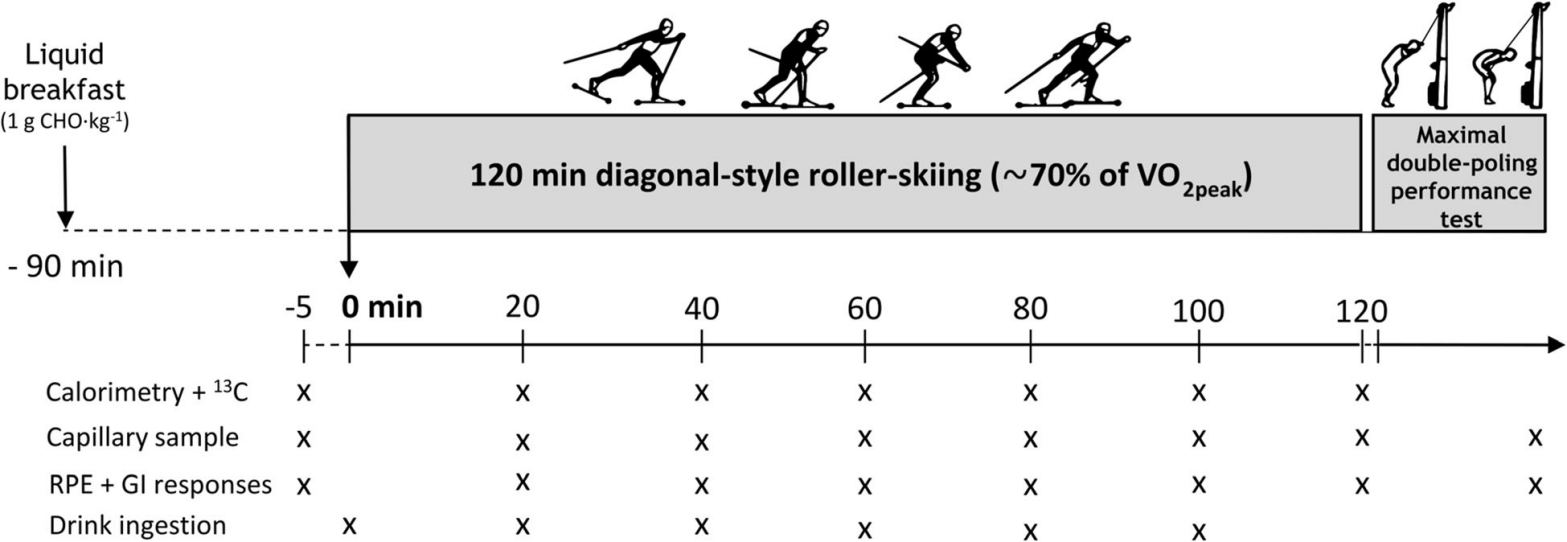
Schematic of the experimental trial day. CHO = carbohydrate, RPE = rating of perceived exertion, GI = gastrointestinal

A schematic of the procedures carried out during the experimental trials is presented in Fig. 1. In accordance with daily CHO needs suggested for rest days and low-intensity activities, as well as acute pre-exercise CHO recommendations [25], participants were provided with individualized meal plans to achieve an intake of 4 g of CHO per kg body mass (BM) the day before the experimental trials. The same individual plan was followed the day prior to both experimental trials and products containing corn or sugar cane were not included in order to reduce background enrichment of expired CO_2_ from naturally derived ^13^C. On the day of the experimental trial, 90 min prior to commencing exercise, 1 g CHO·kg^− 1^ BM (725 ± 100 mL of apple juice [Bravo, Skånemejerier, Sweden]) was provided to the participant.

Athletes arrived at the laboratory at a standardized time (either 06:00 or 09:00) for their two experimental trials, which were separated by 6 ± 1 (mean ± SD) days. Upon arrival at the laboratory, BM was recorded (Seca 764, Hamburg, Germany) and after resting in a seated position for ~ 5 min, a fingertip blood sample was collected for the subsequent analysis of glucose and lactate concentrations (Biosen C-line, EKF diagnostic GmbH, Magdeburg, Germany). Participants then entered the environmental chamber (−5.0 ± 0.2 °C; Kestrel 5500 Weather Meter, Nielsen-Kellerman Company, Boothwyn, PA, USA), where they received their first drink (220 mL of CHO-HG or PLA) before the onset of exercise (time = 0 min).

The CHO-HG drink provided 2.2 g of CHO·min^− 1^ (132 g·h^− 1^) in a ratio of 1:0.8 maltodextrin:fructose and had an osmolality of 750 mOsm·kg^− 1^. Each serving (~ 220 mL) contained 200 mL of water, 44 g of CHO, 0.3 g of NaCl, 0.3 g of sodium alginate and 0.2 g of pectin. In the PLA drink, the CHO was replaced by 0.92 g of erythritol and 20 mg of sweetener (sodium sacharinate, sucralose, L-leucin) per serving, while the amount of fluid, salt and gelling components (i.e., water, NaCl, sodium alginate and pectin) remained the same. Both the CHO-HG and PLA drinks were supplied by Maurten AB (Gothenburg, Sweden), and *in vitro* tests in simulated gastric acid confirmed gelation of both solutions. The maltodextrin (Cargill Nordic A/S) and fructose (Tate & Lyle Sweden AB) were corn-derived with a ^13^C enrichment of − 11.45‰ and − 11.51‰ vs. Pee Dee Bellemnitella (PDB), respectively. The CHO-HG drink was enriched in ^13^C content by adding U-^13^C glucose and U-^13^C fructose (Cambridge Isotope Laboratories, MA, USA) in proportions 1:0.8 and corresponding to 0.487 per mille of the total CHO content. The ^13^C enrichment of the CHO-HG drink reinforced with U-^13^C glucose and U-^13^C fructose was + 28.00‰ vs. PDB.

The 120-min submaximal exercise bout involved diagonal-style roller-skiing and was performed at a constant incline of 5° and a treadmill speed of 9.7 ± 0.2 km·h^− 1^ for the males and 8.5 ± 0.3 km·h^− 1^ for the females. As described for the familiarization trial, treadmill speed was reduced to 4 km^− 1^ every 10 min to allow for a change of sub-technique and movement pattern. In addition, every 20 min, during the 90-s recovery periods, a fingertip blood sample and overall rating of perceived exertion (RPE; Borg category scale 6–20) was collected. Severity of five GI symptoms (gas, nausea, stomach rumbling, urgency to have a bowel movement and abdominal pain) were also rated on a 0–20 scale (0 = no symptoms, 10 = neutral, 20 = worst conceivable symptoms), and a level of digestive comfort was provided (0 = extremely uncomfortable, 10 = neutral, 20 = extremely comfortable) [26]. Following these measurements participants consumed 220 mL of CHO-HG or PLA before the treadmill speed was increased again at the end of the 90-s period.

Following the 120-min submaximal exercise the participants performed a TT, as described for the familiarization trial. Immediately after completion of the TT, subjective RPE, GI symptoms and level of digestive comfort measures were recorded. A fingertip blood sample was collected 3 min after the TT and subsequently analyzed for glucose and lactate concentrations, as described previously. Post-exercise BM was then measured and total loss in BM, used to represent sweat loss and respiratory water losses, was determined by subtracting post-exercise BM from pre-exercise BM. Heart rate was monitored continuously at 5-s intervals throughout the diagonal-skiing and double-poling trials (M400, Polar Electro Oy, Kempele, Finland) and mean values for each minute were subsequently calculated.

### Gas analyses

Expired air was collected during the 120-min submaximal exercise bout in 170-L Douglas bags (C Fritze Consulting, Svedala, Sweden) for 35 s per sample after 17.5 min of each 20-min period (i.e., 2–2.5 min prior to reducing the treadmill speed). After collecting each sample the Douglas bags were immediately removed from the environmental chamber and placed on a bag stand in a thermoneutral room and analyzed the same day, following the exercise trials. The fractional concentrations of O_2_ were determined with an S-3A oxygen analyzer and CO_2_ concentrations were determined with a CD 3-A carbon dioxide analyzer with a P-61B infrared sensor (AEI Technologies Inc., Pittsburgh, PA, USA). Expired gas volume was measured with a 170-L spirometer (Fabri, Spånga, Sweden) with a fast-responding temperature sensor (Greissinger, Würzburg, Germany) attached to the top of the inner cylinder. For the measurement of ^13^C/^12^C in the expired CO_2_, two smaller expired gas samples were drawn from each Douglas bag into 65-mL syringes (Kendall, Monoject, UK) connected via a 3-way valve. The samples were then infused into two 12 mL vials (Labco Ltd., Lampeter, UK) for later analysis.

The breath samples were analyzed for ^13^CO_2_/^12^CO_2_ enrichment (δ^13^C) using a Thermo Scientific Delta Ray isotope ratio infrared spectrometer (IRIS) with a Universal Reference Interface (URI) and a Teledyne CETAC ASX-7100 autosampler. Every two samples were bracketed by calibrating gas (δ ^13^C 27.8 ‰ VPDB). The ^13^C enrichment of beverage content was determined using a Costech Elemental Analyzer (ECS 4010; Costech International, Pioltello, Italy) in continuous flow mode coupled to a Thermo Scientific Delta V plus (ThermoFisher Scientific, Bremen, Germany) isotope ratio mass spectrometer (Friedrich-Alexander-Universität, Erlangen, Germany). All isotope ratio data were normalized to the Vienna Pee Dee Belemnite (VPDB) scale.

### Calculations

Rates of total CHO and fat oxidation (g·min^− 1^) during the submaximal exercise were calculated from $\dot{\mathrm{V}}$O_2_ and $\dot{\mathrm{V}}$CO_2_ (L·min^− 1^) using the following stoichiometric equations, [27] with the assumption that protein oxidation during exercise was negligible:

1 $$ \mathrm{CHO}\ \left(\mathrm{g}\cdotp {\min}^{-1}\right)=\left(4.585\times \dot{\mathrm{V}}{\mathrm{CO}}_2\right)-\left(3.226\times \dot{\mathrm{V}}{\mathrm{O}}_2\right) $$

2 $$ \mathrm{Fat}\ \left(\mathrm{g}\cdotp {\min}^{-1}\right)=\left(1.695\times \dot{\mathrm{V}}{\mathrm{CO}}_2\right)-\left(1.701\times \dot{\mathrm{V}}{\mathrm{O}}_2\right) $$

The isotopic enrichment of the ingested glucose and fructose was expressed as the ‰ difference between the δ^13^C/^12^C ratio of the sample and a known laboratory reference standard [28]:

3 $$ {\updelta}^{13}\mathrm{C}=\left(\left(\frac{13C/12C\  sample}{13C/12C\  standard}\right)-1\right)\cdotp {10}^3 $$

The δ^13^C was then related to an international standard (VPDB). In the CHO-HG trial, the rate of exogenous oxidation was calculated using the formula of Mosora et al. [29]:

4 $$ \mathrm{Exogenous}\ \mathrm{CHO}\ \mathrm{oxidation}\ \left(\mathrm{g}\cdotp {\mathit{\min}}^{-1}\right)={VCO}_2\times \left(\frac{\updelta \mathrm{Exp}-{\updelta \mathrm{Exp}}_{bkg}}{\delta Ing-{\updelta \mathrm{Exp}}_{bkg}}\right)\left(\frac{1}{k}\right) $$

where δExp is the ^13^C enrichment of expired CO_2_ during exercise, δIng is the ^13^C enrichment of the CHO-HG solution, δExp_bkg_ is the ^13^C enrichment of expired air in the PLA trial and *k* (0.7467) is the amount of CO_2_ (L·min^−1^) produced for the complete oxidation of 1 g of glucose. A methodological limitation when calculating exogenous CHO oxidation rates from expired ^13^CO_2_ is the retention of ^13^CO_2_ in the circulating bicarbonate pool [30]. To take this slow equilibration process into account, and hence the delayed appearance of ^13^C in the breath, the computations were only made during the last 60 min of exercise.

### Statistical analysis

All data were checked for normality using the Shapiro-Wilk test. Independent *t*-tests were used for between-group comparisons (e.g., sex differences), while paired samples *t-*tests were used for within-group comparisons (e.g., TT performance and post-TT measurements). A two-way analysis of variance (ANOVA) with repeated measures was performed on all participants (*n* = 12) to assess differences in breath ^13^C enrichment, RER, substrate oxidation, blood markers and perceptual variables (e.g., RPE and GI symptoms) over time between the two trials (CHO-HG and PLA). A three-way mixed design factorial ANOVA considering time × trial × sex was used to identify differences in metabolic and perceptual variables. Substrate oxidation rates are, unless stated otherwise, expressed as a percent of LBM (g·min^− 1^·kg LBM^− 1^·10^− 2^). Total CHO (CHO_total_), exogenous CHO (CHO_exo_), endogenous CHO (CHO_endo_) and fat (FAT) oxidation rates, as well as RER, were calculated over the last 60 min of exercise. Breath ^13^C enrichment, blood glucose and lactate concentrations, RPE and GI symptoms were calculated over the whole 120-min exercise bout, including pre-exercise (at rest). Bonferroni post-hoc adjustments were used to identify the location of significant differences when the ANOVA yielded a significant *F* ratio. Analyses were adjusted by use of the Greenhouse-Geisser correction where necessary. Partial Eta-squared (_p_η^2^) was calculated as a measure of effect size for the ANOVA, where values of 0.01, 0.06, and 0.15 were considered as small, medium, and large, respectively [31]. Cohen’s *d* (*d*) was calculated as a measure of effect size for pairwise comparisons, where values of 0.2, 0.5, and 0.8 were considered as small, medium, and large, respectively [31]. Results are presented as mean ± SD and statistical significance was set at *P* < 0.05. All statistical analyses were conducted using SPSS for Windows version 25 (Chicago, Illinois, USA).

## Results

### Blinding success

Six of the 12 participants correctly guessed the drink solutions (CHO-HG and PLA), while the remaining six guessed incorrectly.

### Submaximal exercise

#### Exercise intensity and energy expenditure

Relative exercise intensity during the 120-min submaximal exercise bout was 69.3 ± 2.9% of $\dot{\mathrm{V}}$O_2_peak (80.4 ± 3.9% of HR_max_)_,_ and was not different for trial (*P* = 0.824, _p_η^2^ = 0.005) or sex *(P* = 0.507, _p_η^2^ = 0.045). Due to greater absolute work rates for the males, absolute $\dot{\mathrm{V}}$O_2_ and energy expenditure were significantly higher than for the females (3.7 ± 0.3 vs. 2.6 ± 0.6 L·min^− 1^, *P* < 0.0001, _p_η^2^ = 0.778, and 18.5 ± 1.5 vs. 13.2 ± 1.6 kcal·10^− 1^, *P* < 0.001, _p_η^2^ = 0.790, respectively). However, when adjusting for LBM the differences between males and females for $\dot{\mathrm{V}}$O_2_ (1.8 mL·min^− 1^; 95% confidence interval [CI] = − 2.3 to 6.0 mL·min^− 1^, *P* = 0.354, _p_η^2^ = 0.086) and energy expenditure (1.3 kcal· min^− 1^; 95% CI − 1.1 to 3.7 kcal · min^− 1^, *P* = *0.365,*_p_η^2^ = 0.128) were not significant.

#### Breath enrichment

**Fig. 2:**
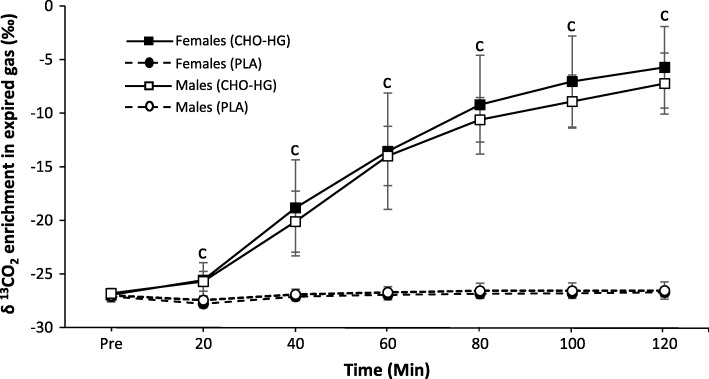
Mean ± SD changes in δ ^13^CO2 in expired CO_2_ during the 120-min submaximal exercise bout. ^c^Significant difference between CHO-HG and PLA (*P* < 0.0001; *n* = 12)

Changes in expired-air δ ^13^CO_2_ during CHO-HG and PLA are shown in Fig. 2. No differences were observed at rest (− 27.0 ± 0.5 ‰ δ ^13^C vs. PDB; pooled data, *n* = 24). In CHO-HG, there was a significant increase (*P* < 0.0001, _p_η^2^ = 0.96) in breath ^13^CO_2_ enrichment over time, reaching − 6.5 ± 3.3 ‰ δ ^13^C vs. PDB after 120 min (*n* = 12). During the PLA trial, expired breath ^13^C enrichment remained largely unchanged and was significantly lower than the CHO-HG trial throughout exercise (*P* < 0.0001, _p_η^2^ = 0.955).

#### Substrate oxidation

**Fig. 3:**
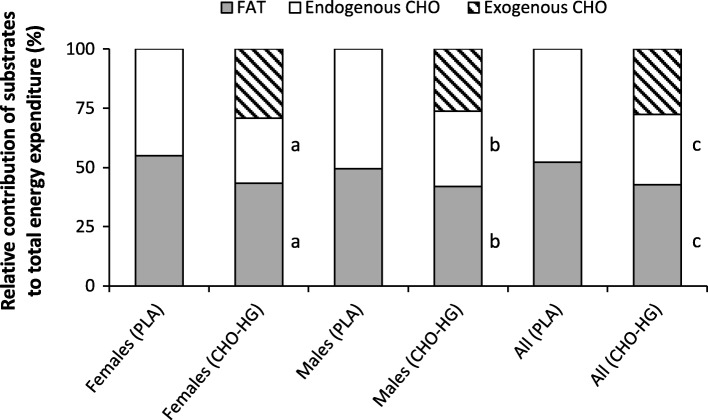
The relative contributions of fat, endogenous carbohydrate and exogenous carbohydrate oxidation to the total energy yield during the second hour of exercise (60–120 min). PLA, placebo trial; CHO-HG, carbohydrate trial. Significant difference between CHO-HG and PLA (*P* < 0.05) for ^a^females (*n* = 6) and ^b^males (*n* = 6) and ^c^all athletes (*n* = 12)

Mean RER values and total substrate oxidation rates during the last hour (60–120 min) of exercise are presented in Table 2. In response to CHO-HG ingestion, FAT oxidation was significantly lower and CHO_total_ oxidation was significantly higher than in PLA. In addition, while CHO_exo_ oxidation increased over time in CHO-HG, CHO_endo_ oxidation decreased. Mean and individual CHO_exo_ oxidation rates for females and males during submaximal exercise in the CHO-HG trial can be viewed in a supplementary figure (Additional file 1). The relative contributions of FAT, CHO_endo_ and CHO_exo_ oxidation to the total energy yield are shown in Fig. 3. Similar reductions in relative contributions from FAT and CHO_endo_ oxidation were observed with CHO-HG compared to PLA for females and males (9.5 ± 4.8% and 18.1 ± 6.4% for FAT and CHO_endo_ oxidation, respectively; *n* = 12, *P* < 0.001; *d* > 1.01). Peak CHO_exo_ reached 1.33 ± 0.27 g·min^− 1^ at 120 min (n = 12) and there was a tendency for males to oxidize more CHO_exo_ during the last 60 min of exercise than the females (mean difference 0.27 g·min^− 1^, 95% CI = − 0.019 to 0.56, *P* = 0.064, _p_η^2^ = 0.303). Between-group comparisons showed no significant main effects of sex for RER or the absolute (Table 2) or relative (Fig. 3) contributions of FAT, CHO_total_, CHO_endo_ or CHO_exo_ oxidation during the last 60 min of exercise.

**Table 2:** Mean ± SD RER and substrate oxidation (g min^− 1^·kg LBM^− 1^·10^− 2^) during the second hour of submaximal exercise (60–120 min)

	Females (*n* = 6)		Males (*n* = 6)		All (*n* = 12)		Two-way ANOVA *P* value (_p_η^2^)		
	PLA	CHO-HG	PLA	CHO-HG	PLA	CHO-HG	Time	Trial	Interaction
RER	0.83 ± 0.02	0.87 ± 0.02**	0.85 ± 0.01	0.87 ± 0.01*	0.84 ± 0.01	0.87 ± 0.01	0.831 (0.011)	**< 0.0001** (0.813)	**0.042** (0.217)
FAT	95.4 ± 8.3	75.7 ± 7.2**	90.0 ± 9.5	77.4 ± 5.5*	92.6 ± 8.9	76.6 ± 6.2	0.749 (0.019)	**< 0.0001** (0.833)	**0.040** (0.220)
CHO_total_	199.1 ± 33.0	250.7 ± 30.8*	231.4 ± 26.5	262.4 ± 30.5*	215.2 ± 31.2	256.5 ± 29.9	0.875 (0.009)	**< 0.0001** (0.772)	0.089 (0.177)
CHO_exo_	–	128.3 ± 30.2	–	122.1 ± 17.9	–	125.2 ± 23.9	**< 0.0001** (0.792)	–	–
CHO_endo_	199.1 ± 33.0	122.4 ± 34.7**	231.4 ± 26.5	140.3 ± 33.0*	215.2 ± 31.2	131.4 ± 33.6	**0.013** (0.347)	**< 0.0001** (0.909)	**0.013** (0.275)

Significant *P < 0.05 and **P < 0.0001 main effect of trial (PLA vs. CHO-HG) for within-group comparisons (e.g., females and males, respectively); *ANOVA* analysis of variance, _*p*_*η*^*2*^ partial Eta-squared, *PLA* placebo, *CHO-HG* carbohydrate-hydrogel, *RER* respiratory exchange ratio, *FAT* fat oxidation, *CHO*_*total*_ total carbohydrate oxidation, *CHO*_*endo*_ endogenous carbohydrate oxidation, *CHO*_*exo*_ exogenous carbohydrate oxidation. Significant *P*-value (< 0.05) is displayed in bold

#### Blood metabolites

**Fig. 4:**
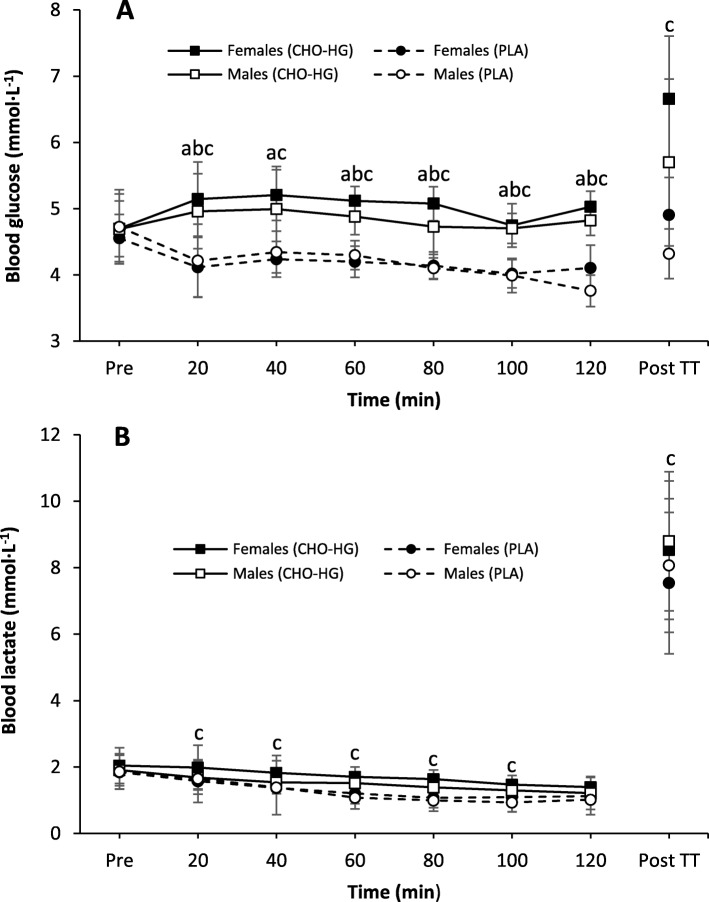
Mean ± SD blood glucose (**a**) and lactate (**b**) concentrations. Significant difference between CHO-HG and PLA for ^a^females (*n* = 6) and ^b^males (*n* = 6) and ^c^all athletes (*n* = 12)

Blood glucose and lactate concentrations are shown in Fig. 4. Following the onset of exercise blood glucose concentrations were consistently higher in CHO-HG compared with PLA (*P* < 0.0001, _p_η^2^ = 0.877). In addition, blood lactate concentrations decreased over time in both conditions (*P* < 0.0001, _p_η^2^ = 0.606) and were significantly higher in CHO-HG compared to PLA (*P* < 0.0001, _p_η^2^ = 0.687). No differences were observed between the sexes for blood glucose or lactate concentrations during submaximal exercise.

#### Perceptual variables

No differences were observed between trials for any of the GI discomfort variables or RPE at rest. Following the onset of exercise, RPE gradually increased (main effect of time: *P* < 0.0001, _p_η^2^ = 0.435) from 12.7 at 20 min (95% CI = 12.0 to 13.3) to 13.6 at 120 min (95% CI = 13.2 to 14.0), with no significant difference between CHO-HG and PLA (Table 3). Level of digestive comfort gradually decreased (main effect of time: *P* < 0.019, _p_η^2^ = 0.311) from 16.1 (95% CI = 14.2 to 18.1) at rest to 14.1 (95% CI = 12.6 to 15.7) at 120 min. As demonstrated in Table 3, mean ratings of GI discomfort symptoms were generally low. However, one participant rated “abdominal pain” and “nausea” as 14 (where a rating of 10 is “neutral” and 20 reflects “worst conceivable symptoms”) during the last hour of exercise in the CHO-HG trial. No sex differences were observed for RPE or GI symptoms.

**Table 3:** Ratings of perceived exertion (RPE) and perceptions of gastrointestinal symptoms during the 120-min submaximal exercise (*n* = 12)

	PLA		CHO-HG		Two-way ANOVA *P* value (_p_η^2^)		
	Mean ± SD	Range (min–max)	Mean ± SD	Range (min–max)	Time	Trial	Interaction
RPE	13.2 ± 0.9	11–16	13.3 ± 0.8	10–15	**< 0.0001** (0.435)	0.777 (0.008)	0.205 (0.120)
Gas^a^	2.5 ± 2.7	0–11	3.4 ± 3.0	0–12	**0.014** (0.307)	0.110 (0.215)	0.235 (0.124)
Nausea^a^	1.6 ± 2.2	0–10	2.2 ± 3.2	0–14	0.135 (0.171)	0.511 (0.040)	0.681 (0.028)
Stomach rumbling^a^	2.7 ± 1.9	0–8	2.2 ± 2.0	0–7	0.069 (0.205)	0.182 (0.156)	**0.042** (0.185)
Abdominal pain^a^	2.5 ± 2.7	0–11	3.5 ± 3.3	0–14	**0.009** (0.376)	0.282 (0.104)	0.258 (0.116)
Urgency to have a bowel movement^a^	2.7 ± 3.0	0–12	2.4 ± 2.7	0–10	**0.017** (0.336)	0.394 (0.067)	0.886 (0.016)
Level of digestive comfort^b^	14.6 ± 3.0	8–19	14.9 ± 2.7	9–20	**0.011** (0.360)	0.656 (0.019)	0.482 (0.061)

^a^0 = no symptoms, 20 = worst conceivable symptoms; ^b^0 = extremely uncomfortable, 20 = extremely comfortable (10 = neutral); _*p*_*η*^*2*^ partial Eta-square. Significant *P*-value (< 0.05) is displayed in bold

#### Relative changes in BM

The relative change in BM (as a % of total BM) was not significantly different between trials, although there was a trend for CHO-HG to maintain euhydration more than with PLA (0.1 ± 0.7% versus − 0.3 ± 0.7%, respectively; *P* = 0.068, *d* = 0.0061). However, the males lost 0.4 ± 0.6% of BM while the females gained 0.4 ± 0.5% in CHO-HG (*P* = 0.036). A similar tendency was observed in PLA, where the males lost 0.7 ± 0.5% and the females gained 0.02 ± 0.7% (*P* = 0.051).

### Time trial (TT)

#### Performance

**Fig. 5:**
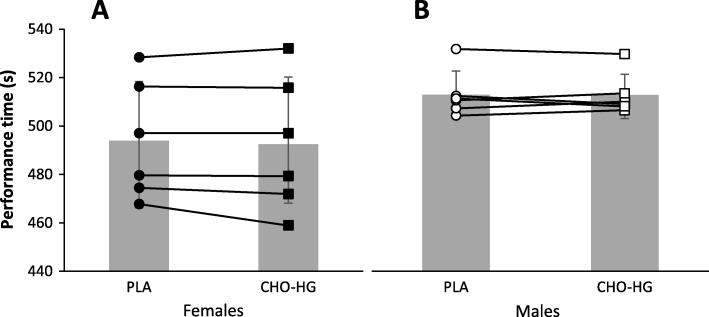
Mean ± SD time taken to complete the 2000-m TT for females (**a**) and 2400-m TT for males (**b**). PLA, placebo trial; CHO-HG, carbohydrate trial

Average power output for CHO-HG and PLA was 239 ± 16 W and 238 ± 16 W, respectively (mean difference 1.3 ± 5.4 W; 95% CI = − 2.1 to 5.4 W, *P* = 0.411, *d* = 0.0061). Consequently, the difference in TT times between CHO-HG and PLA (Fig. 5) was not significant (0.8 ± 3.5 s; 95% CI = − 3.0 to 1.5 s, *P* = 0.461, *d* = 0.035). When the total TT distance was divided into five time splits (i.e., time [s] to complete each of the successive 5 × 400 m and 480 m splits for the females and males, respectively), there was a significant effect of time (i.e., pacing; *P* < 0.0001, _p_η^2^ = 0.678). However, there was no significant interaction effect between time and trial (*P* = 0.173, _p_η^2^ = 0.162). The relative difference in TT performance between CHO-HG and PLA, independent of flywheel resistance and TT distance, was not significantly different for the males versus females (− 0.3% ± 3.5%; 95% CI = − 1.3 to 0.7%, *P* = 0.495, *d* = 0.157).

#### Blood metabolites

Post-TT blood glucose and lactate concentrations, presented in Fig. 4, were significantly higher in CHO-HG compared to PLA (*P* = 0.0007, *d* = 1.704 and *P* = 0.022, *d* = 0.434 for glucose and lactate, respectively). There was a tendency for glucose concentrations to be higher in the females compared to the males in PLA (*P* = 0.061, *d* = 1.220), but not in CHO-HG (*P* = 0.169, *d* = 0.857).

#### Perceptual variables

Mean RPE following the TT was not significantly different for CHO-HG compared to PLA (18.2 ± 1.4 versus 18.3 ± 1.1; *P* = 0.62, *d* = 1.307). All ratings of GI discomfort following the TT were low in both CHO-HG and PLA (mean < 3.8, range 0–11) and mean level of digestive comfort was 14.3 in both trials (95% CI = 12.7 to 15.9, *P* = 0.90, *d* = 0.003). The mean ± SD and range of responses for RPE, GI discomfort and level of digestive comfort reported after the TT are presented in a supplementary table (Additional file 2).

## Discussion

The present study demonstrated that frequent ingestion of CHO during 120 min of moderate-intensity diagonal roller-skiing did not enhance performance during a subsequent self-paced, double-poling time-trial in elite XC ski athletes. This finding was despite significantly higher endogenous CHO oxidation during the PLA trial, in comparison to the CHO-HG trial, and indicates that stored muscle and liver glycogen were not depleted after 2 h of exercise at ~ 70% of $\dot{\mathrm{V}}$O_2_peak (~ 80% of HR_max_) in these individuals. Despite the high ingestion rate (2.2 g CHO·min^− 1^) of a highly concentrated (18%) maltodextrin-fructose mixture including gelling components, no severe GI symptoms were reported during submaximal exercise or following maximal exercise in the CHO-HG trial. Regarding sex comparisons, no differences were identified between the females and males for substrate utilization, RPE, GI outcomes or TT performance. However, the males performed more absolute work, had a higher absolute $\dot{\mathrm{V}}$O_2_ and energy expenditure and experienced a greater reduction in BM compared with the females.

### Substrate utilization and metabolism during the submaximal exercise

It is well established that compared with a control (i.e., a placebo or water), exogenous CHO provision during prolonged exercise increases total CHO oxidation, decreases fat oxidation and reduces the oxidation of endogenous CHO, and that these alterations in substrate metabolism are entirely attributed to the oxidation of ingested (i.e., exogenous) CHO [21–23]. Further, in contrast to glucose-only feedings during exercise, blood lactate concentration is known to increase in response to fructose ingested within multiple-transportable CHO solutions [3, 32]. The results from the current study support these previous findings, whereby CHO-HG ingestion led to significant increases in blood lactate concentration and total and exogenous CHO oxidation, as well as decreases in endogenous CHO and fat oxidation, in comparison to the PLA trial.

Consuming 2.2 g·min^− 1^ of a 0.8:1 maltodextrin:fructose hydrogel solution in the current study led to a peak exogenous CHO oxidation rate of 1.33 g·min^− 1^ (range 0.89–1.66 g·min^− 1^) after 120 min. Ingesting 2.4 g·min^− 1^ of a 1:1 glucose:sucrose solution (i.e., 1:0.3 glucose:fructose) has previously been demonstrated to elicit a mean peak oxidation rate of 1.20 g·min^− 1^ at the end of 120 min of exercise at ~ 63% of $\dot{\mathrm{V}}$O_2_max among cyclists [4]. Two related studies demonstrated peak oxidation rates of 1.70 and 1.75 g·min^− 1^, respectively, after 150 min of exercise at ~ 60–62% of $\dot{\mathrm{V}}$O_2_max when ingesting glucose and fructose at 1:0.6 and 1:1 ratios, respectively [3, 32]. Differences in exogenous oxidation rates between the current study compared with those previously reported by Jentjens and colleagues likely resides from differences in the experimental protocols (i.e., amount and type of CHO ingested, and exercise duration). Furthermore, with no plateau observed towards the end of exercise, it may be assumed that the peak exogenous CHO oxidation rate would have exceeded 1.33 g·min^− 1^ in the present study if the submaximal exercise bout had continued beyond 120 min.

### Carbohydrate-hydrogel ingestion and gastrointestinal symptoms

Gastrointestinal discomfort is considered to be a limiting factor in moderate- to high-intensity exercise (i.e., ≥ 60% $\dot{\mathrm{V}}$O_2_max) lasting ≥ 2 h, and symptoms might be further exacerbated by dehydration and excessive CHO intake [2, 33]. In order to test the potential effects of CHO-HG on GI symptoms, and concomitantly maximize CHO_exo_ oxidation, a CHO ingestion rate of 2.2 g·min^− 1^ was selected in the present study. This is in excess of current CHO intake guidelines, which recommend up to 1.5 g·min^− 1^ [2]. The CHO solution provided the participants with a similar amount of fluid (i.e., 600 mL·h^− 1^) previously shown to be ingested during competition by elite XC ski athletes in cold conditions [34]. Despite the high CHO concentration (18%), no differences in GI discomfort or level of digestive comfort were observed in CHO-HG compared to PLA. These findings might be due to the cold ambient conditions during the 120-min submaximal exercise, which has been shown to decrease the incidence and severity of GI symptoms compared to hot conditions [33]. Furthermore, mechanical causes of GI symptoms, such as shaking of the intra-abdominal contents, would likely be reduced during XC skiing compared to running, for example. Thus, future research efforts might address whether the high GI tolerability observed following CHO-HG ingestion can be attributed to the added gelling polysaccharides per se. As well as delineating specific mechanisms on the gastric and intestinal behaviors in response to hydrogel exposure, comparisons with an isocaloric CHO-only control intake under gut-challenging prolonged, high-intensity exercise in different ambient conditions is warranted.

### Carbohydrate-hydrogel ingestion and time-trial performance

Contrary to the hypothesis, no ergogenic effect was observed for TT performance following CHO ingestion in the current study. This is in contrast to most [35, 36], but not all [37], previous placebo-controlled CHO studies employing similar protocols in terms of the duration and intensity of submaximal exercise (e.g., 105–120 min at ~ 70% $\dot{\mathrm{V}}$O_2_max) and subsequent performance tests (e.g., lasting 8–15 min). Plausible explanations for this discrepancy may relate to differences in the exercise modalities used and the training status of the participants. For example, diagonal XC skiing was used in the present study, which, in contrast to the majority of previous studies where cycling protocols have been employed, involves whole-body exercise with the upper body generating ~ 50–75% of the propulsive power output during moderately-intense exercise [38]. Since a substantial portion of the work done to propel the XC skier forwards during the submaximal exercise bout would have been performed by the lower-body, it is possible that endogenous CHO availability was still adequate in the upper-body musculature in PLA to meet the high energy demands of the subsequent ~ 8.4-min double-poling time-trial. That the power output profile patterns did not differ between PLA and CHO-HG, including an increase in power output during the final 20% of the total TT distance (possibly relating to an anaerobic energy reserve), supports this contention that CHO would have still been locally available in the upper body even towards the end of the TT in the PLA trial. However, although muscles of the upper limbs have been shown to be the primary working muscles involved in double poling at lower-exercise intensities, an increasing involvement of the torso, hip and leg muscles is evident at higher exercise intensities [39].

Although muscle glycogen content was not measured in the current study, it is possible that the submaximal exercise was not demanding enough to deplete endogenous glycogen stores in this specific group of elite athletes. A recent meta-analysis [40] of skeletal muscle glycogen utilization concluded that ~ 120 min of exercise at 70% of $\dot{\mathrm{V}}$O_2_max initiated with normal muscle glycogen content (i.e., 400 mmol∙kg^− 1^ dry weight [d.w.]) would lead to the attainment of critical muscle glycogen levels (i.e., 250–300 mmol∙kg^− 1^ d.w.), which have been associated with reductions in peak power output [9]. However, the majority of participants in the present study were world-class endurance athletes and would likely have a far superior capacity for oxidizing fat and sparing CHO when exercising for a prolonged period of time at this intensity [41]. Future research investigating the impact of CHO supplementation on performance and determinants of fatigue related to substrate utilization during XC skiing with elite skiers and biathletes should aim to increase the duration and/or intensity of the submaximal preload. Moreover, including a TT with the same sub-technique and/or repeated bouts of high-intensity exercise would also allow for a closer simulation of real-world competition demands.

### Sex comparisons and substrate utilization

As well as being the first study to investigate exogenous CHO oxidation during XC skiing (i.e., whole-body exercise), the present study is also the first to examine sex differences when ingesting a multiple-transportable CHO solution during exercise. Compared to PLA, CHO-HG ingestion reduced the reliance on endogenous CHO oxidation over the final hour of exercise in both sexes by ~ 18%, which is comparable to reductions previously observed for females and males (~ 15%) in two studies using high ingestion rates (1.5–2 g·min^− 1^) of glucose only [22, 23]. In the present study, endogenous CHO oxidation contributed ~ 28 and 32% to the total energy yield in the CHO-HG trial for females and males, respectively. In contrast, Riddell et al. [21] showed that the relative endogenous CHO oxidation to the total energy yield was significantly higher in females (~ 14%) than in males (~ 5%). However in that study the ingestion rate was based on BM (1 g glucose·kg BM·h^− 1^), resulting in ~ 1.0 and 1.3 g CHO·min^− 1^ for the females and males, respectively. On balance, the current and previous studies suggest that when ingesting the same absolute amount of a single- or multiple-transportable CHO, the relative reduction in endogenous CHO oxidation to total energy contribution appears to be similar between the sexes.

Regarding exogenous CHO oxidation, the current and previous studies [20–23] indicate that the relative contribution to total energy expenditure is consistently, although not necessarily significantly, ~ 2–4% higher in females than in males. However, when expressed in absolute terms (g·min^− 1^), sex differences in exogenous CHO oxidation have showed mixed results. M’Kaouar et al. [20] reported that females oxidized ~ 33% less exogenous CHO compared with males (~ 0.6 versus 0.9 g·min^− 1^) during 120 min of cycling exercise at ~ 65% of $\dot{\mathrm{V}}$O_2_max. By contrast, other studies have shown no significant sex differences in absolute exogenous CHO oxidation when cycling for 90–120 min at 57–67% of $\dot{\mathrm{V}}$ O_2_max [21–23]. The females in the current study tended (*P* = 0.064) to oxidize ~ 20% less exogenous CHO than the males during the last hour of exercise (~ 1.2 versus 1.5 g·min^− 1^). However, the novel data presented in the current study have demonstrated that females have the capacity to substantially increase CHO_exo_ oxidation when fed a multiple-transportable CHO solution at a high ingestion rate, with observed peak oxidation rates up to 1.61 g CHO·min^− 1^, well in excess of SGLT1 transporter saturation (i.e., ~ 1 g·min^− 1^). Due to the small sample sizes used in the current and previous studies (i.e., *n* = 6–8), further research employing larger samples is necessary to assess whether there is indeed a sex difference in exogenous CHO oxidation following the ingestion of multiple-transportable CHO solutions.

### Strengths and limitations

The novel approaches and strengths of this study include the use of an innovative multiple-transportable carbohydrate hydrogel during exercise under conditions where energy requirements (CHO in particular) are expected to be high, and sweat rates low (e.g., whole-body exercise in the cold). Moreover, the involvement of a familiarization trial and the standardized dietary preparation ensured that conditions were controlled between participants and trials. Perhaps most noteworthy, though, is the unusually high level of the participating athletes, most of whom were world-class (with half winning Olympic and World Championship medals in the year of data collection), as well as the mixed-sex nature of the sample.

A number of limitations in the study design should, however, be acknowledged. For example, a CHO control without additional gelling polysaccharides was not administered, and neither was a non-polysaccharide placebo. This was due to the nature of the sample group (i.e., a national team in preparation for an Olympic Games only 6 months away), so prescribing additional long-duration and highly-controlled trials was not possible. Therefore, the experimental solution (CHO-HG) and a placebo with gelling agents but no CHO were prioritized. In addition to this, and a low within-sex sample size, the menstrual phase of the female participants (which may influence substrate oxidation) was not controlled for. However, while ovarian hormones might affect metabolic regulation during exercise [16] results are conflicting [15] and variability in substrate metabolism seems more likely due to between- and within-subject variations than the menstrual-cycle phase. Furthermore, participants were provided with pre-exercise CHO, which has previously been shown to negate the effects of menstrual cycle phase on glucose kinetics by reducing the demand on endogenous glucose production [42]. Three out of six female participants in this study were using hormonal contraceptives, which have also been suggested to alter fat and CHO metabolism during exercise [43]. However, the evidence for this is unclear as no differences in fuel utilization during prolonged exercise were observed between females taking and not taking oral contraceptives [22].

## Conclusions

The present study has shown that ingesting a relatively highly-concentrated maltodextrin-fructose CHO solution with unique gelling properties during prolonged, moderately-intense whole-body exercise (i.e., diagonal XC skiing at ~ 70% of $\dot{\mathrm{V}}$O_2_peak) in a cold environment does not provide an ergogenic effect on subsequent maximal upper-body performance in temperate environmental conditions. The tolerability of the 18% multiple-transportable CHO-HG solution was nevertheless good and did not differ from a placebo control. Compared to traditional sport-drink formulations with CHO concentrations ≤8%, higher concentrations might offer a practical solution to achieving CHO recommendations during prolonged exercise without consuming large fluid volumes, particularly in environmental conditions where sweat rates are expected to be low. A key novel finding of the present study is that exogenous CHO oxidation rates for females can reach well in excess of 1 g CHO·min^− 1^ following intake of a high-energy multiple-transportable CHO solution, which is similar to findings that have been presented repeatedly for males. However, the tendency for females to elicit lower exogenous CHO oxidation rates than males warrants further research, as any potential sex differences in exogenous CHO oxidation following multiple-transportable CHO intake may have implications with respect to CHO-specific recommendations for females and males performing endurance exercise.

## Supplementary information

**Additional file 1. **Exogenous carbohydrate oxidation during exercise in the CHO-HG (carbohydrate-hydrogel) trial in (A) females (*n* = 6) and (B) males (*n* = 6). The thick black line represents the group mean and the thin black lines represent individual responses.

**Additional file 2. **Ratings of perceived exertion (RPE) and perceptions of gastrointestinal symptoms after the double-poling time-trial (*n* = 12).

## Data Availability

All data generated or analyzed during this study are included in this published article (and its supplementary information files).

## Abbreviations

ANOVA: Analysis of variance
BM: Body mass
CHO: Carbohydrate
CHO_endo_: endogenous carbohydrate oxidation
CHO_exo_: exogenous carbohydrate oxidation
CHO-HG: Carbohydrate hydrogel
CHO_total_: total carbohydrate oxidation
GI: Gastrointestinal
HR_max_: maximal heart rate
PLA: Placebo
RPE: Rating of perceived exertion
TT: Time-trial
XC: Cross-country
