# Supplementary material for: Effects of supplementing with an 18% carbohydrate-hydrogel drink versus a placebo during whole-body exercise in −5 °C with elite cross-country ski athletes: a crossover study
Source: J Int Soc Sports Nutr. 2019 Oct 26;16:46. doi: 10.1186/s12970-019-0317-4 (PMC6815417; doi:10.1186/s12970-019-0317-4)
Supplement: Supplementary file 1 — Additional file 1. Exogenous carbohydrate oxidation during exercise in the CHO-HG (carbohydrate-hydrogel) trial in (A) females (n = 6) and (B) males (n = 6). The thick black line represents the group mean and the thin black lines represent individual responses. [file 12970_2019_317_MOESM1_ESM.pdf]

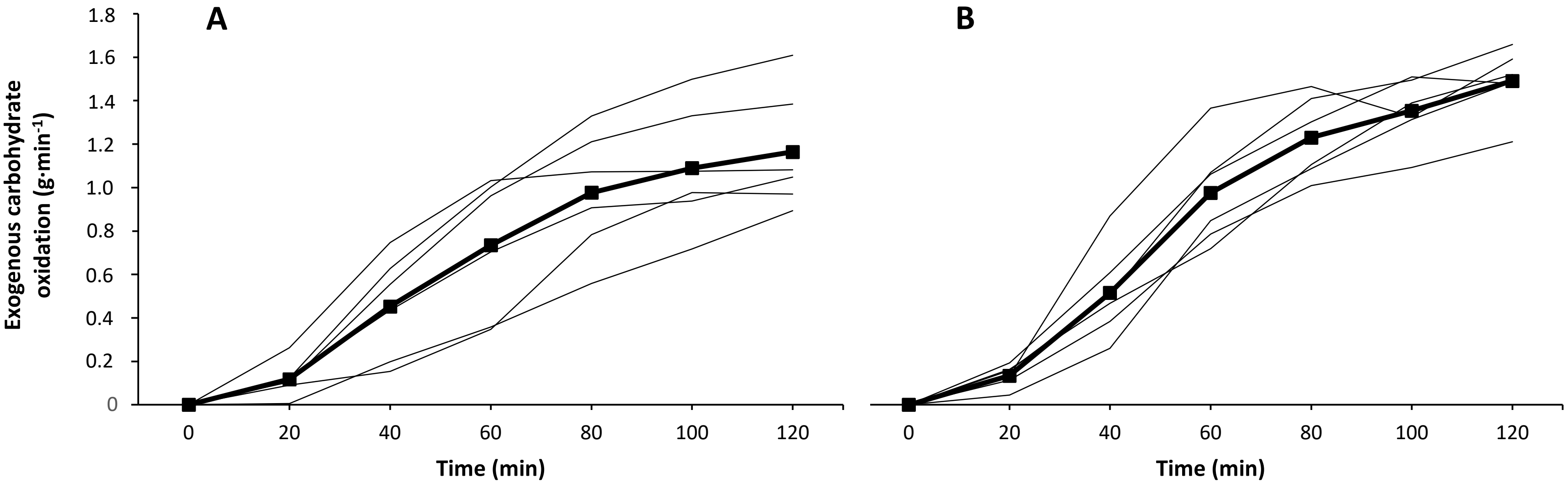

Exogenous carbohydrate oxidation during exercise in the CHO-HG (carbohydrate-hydrogel) trial in (A) females (n=6) and (B) males (n=6). The thick black line represents the group mean and the thin black lines represent individual responses
