# Supplementary material for: Effects of supplementing with an 18% carbohydrate-hydrogel drink versus a placebo during whole-body exercise in −5 °C with elite cross-country ski athletes: a crossover study
Source: J Int Soc Sports Nutr. 2019 Oct 26;16:46. doi: 10.1186/s12970-019-0317-4 (PMC6815417; doi:10.1186/s12970-019-0317-4)
Supplement: Supplementary file 2 — Additional file 2. Ratings of perceived exertion (RPE) and perceptions of gastrointestinal symptoms after the double-poling time-trial (n = 12). [file 12970_2019_317_MOESM2_ESM.docx]

Ratings of perceived exertion (RPE) and perceptions of gastrointestinal symptoms after the double-poling time-trial (n=12)

|  | PLA | |  | | CHO-HG | |  | | |  |
| --- | --- | --- | --- | --- | --- | --- | --- | --- | --- | --- |
|  | Mean ± SD | Range  (min–max) |  | Mean ± SD | | Range  (min–max) | |  | Paired *t*-test (*d*) | |
| RPE | 18.2 ± 1.4 | 15 – 20 |  | 18.3 ± 1.1 | | 16 – 20 | |  | 0.62 (1.307) | |
| Gas^†^ | 2.2 ± 1.9 | 0 – 5 |  | 2.8 ± 2.4 | | 0 – 9 | |  | 0.27 (0.030) | |
| Nausea^†^ | 2.7 ± 2.8 | 0 – 8 |  | 3.5 ± 4.2 | | 0 – 11 | |  | 0.44 (0.026) | |
| Stomach rumbling^†^ | 3.8 ± 2.5 | 0 – 8 |  | 1.8 ± 1.6 | | 0 – 5 | |  | 0.07 (0.378) | |
| Abdominal pain^†^ | 2.6 ± 2.5 | 0 – 10 |  | 3.3 ± 3.3 | | 0 – 10 | |  | 0.46 (0.026) | |
| Urgency to have a bowel movement^†^ | 2.3 ± 2.7 | 0 – 9 |  | 1.6 ± 1.7 | | 0 – 5 | |  | 0.32 (0.033) | |
| Level of digestive comfort^‡^ | 14.3 ± 2.3 | 9 – 18 |  | 14.3 ± 2.9 | | 10 – 18 | |  | 0.90 (0.003) | |
| PLA: placebo trial; CHO-HG: carbohydrate-hydrogel trial  ^†^0 = no symptoms, 20 = worst conceivable symptoms; ^‡^0 = extremely uncomfortable, 20 = extremely comfortable (10 = neutral); *d,* Cohen's *d*. | | | | | | | | | | |
